# Laparoscopic and Open Repair of an Intercostal Abdominal Hernia

**DOI:** 10.7759/cureus.110183

**Published:** 2026-06-03

**Authors:** Erick R Guerra Bocanegra, Ana P Rábago Jamaica, Hector M Alcaraz Amaya, Farah M Hadad Monsiváis

**Affiliations:** 1 General Surgery, Swiss Hospital, Monterrey, MEX

**Keywords:** abdominal wall defect, abdominal wall surgery, intercostal hernia, intercostal hernia laparoscopic repair, intercostal hernia repair, primary closure of intercostal hernia

## Abstract

Intercostal hernias are uncommon defects of the thoracoabdominal wall.

We present the case of a 53-year-old male patient with progressive bulging of the left lateral abdominal wall. Computed tomography (CT) with the Valsalva maneuver demonstrated disruption of the left external oblique muscle and muscular asymmetry consistent with a hernia defect. Surgical repair was successfully performed using a combined laparoscopic and open approach.

This case highlights the importance of dynamic imaging for diagnosis and the role of an individualized surgical strategy in the management of complex lateral abdominal wall defects.

## Introduction

Intercostal hernias are an uncommon clinical entity within the spectrum of thoracoabdominal wall defects, characterized by the protrusion of intra-abdominal or extraperitoneal contents through defects involving the intercostal spaces or the lateral abdominal musculature [1].

The incidence is extremely low, with fewer than 300 cases reported in the literature, thereby limiting the availability of standardized guidelines for diagnosis and management [2].

From an etiopathogenic standpoint, these hernias may be classified as congenital or acquired, the latter being the most frequent and generally associated with blunt or penetrating trauma, previous surgical procedures, infections, or conditions associated with chronically increased intra-abdominal pressure, such as chronic obstructive pulmonary disease [1].

In particular, defects secondary to abdominal wall or lumbar surgical interventions have been increasingly reported, underscoring the role of myofascial disruption and potential segmental denervation as relevant pathophysiological mechanisms [2].

Anatomically, the lateral abdominal wall contains areas of inherent weakness, particularly within the region extending from the costal margin to the iliac crest, where the arrangement of the oblique and transversus abdominis muscles may become compromised following surgical procedures or trauma. Disruption of the continuity of the external oblique muscle, as observed in the present case, represents a critical factor in the development of hernia defects [2].

Clinical diagnosis may be challenging because of the nonspecific presentation, frequently manifesting as a lateral bulge that may be intermittent or dependent on increases in intra-abdominal pressure. In this context, computed tomography (CT) with the Valsalva maneuver is considered the gold standard, as it allows dynamic assessment of the defect, precise anatomical characterization, and differentiation from other entities such as lumbar hernias, abdominal wall eventration, or denervation-related muscular atrophy [3].

The management of these hernias remains controversial, with strategies ranging from conservative treatment in asymptomatic patients to surgical repair in symptomatic individuals or those at risk of complications [2]. The choice of surgical technique depends on multiple factors, including defect size, surrounding tissue quality, and the presence of neuromuscular alterations [4].

In this report, we present the case of a 53-year-old patient with an intercostal hernia that was successfully repaired using a combined laparoscopic and open surgical approach.

## Case presentation

A 53-year-old male patient with a medical history significant for diabetes mellitus and arterial hypertension, and a prior bilateral inguinal hernia repair performed 13 years earlier, presented for evaluation of progressive enlargement of the left lateral abdominal wall over the preceding six months. The patient denied any known connective tissue disorders.

He reported that the bulge became more prominent during maneuvers associated with increased intra-abdominal pressure, such as prolonged standing or physical exertion, without severe pain, skin discoloration, or associated systemic symptoms.

Physical examination revealed a localized bulge located in the left lateral abdominal wall, soft in consistency, measuring approximately 25 cm in length and 15 cm in width, with poorly defined borders and partial reducibility on palpation. Deep palpation suggested a defect involving the muscular planes of the region, without evidence of an underlying solid mass.

Thoracoabdominal computed tomography complemented with the Valsalva maneuver demonstrated significant thinning of the left external oblique muscle associated with disruption of its lateral insertion, resulting in a structural defect of the thoracoabdominal wall (Figure 1 and Figure 2).

**Figure 1:**
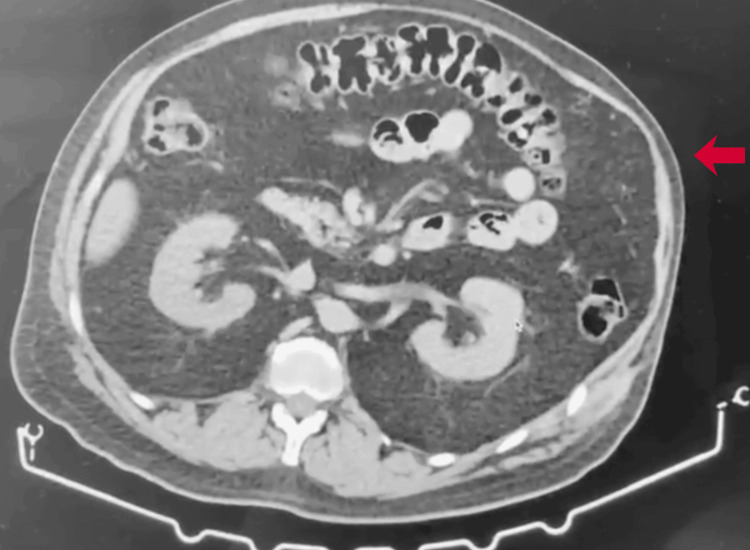
Axial computed tomography image demonstrating thinning and disruption of the left external oblique muscle with protrusion of tissue through the lateral abdominal wall defect

**Figure 2:**
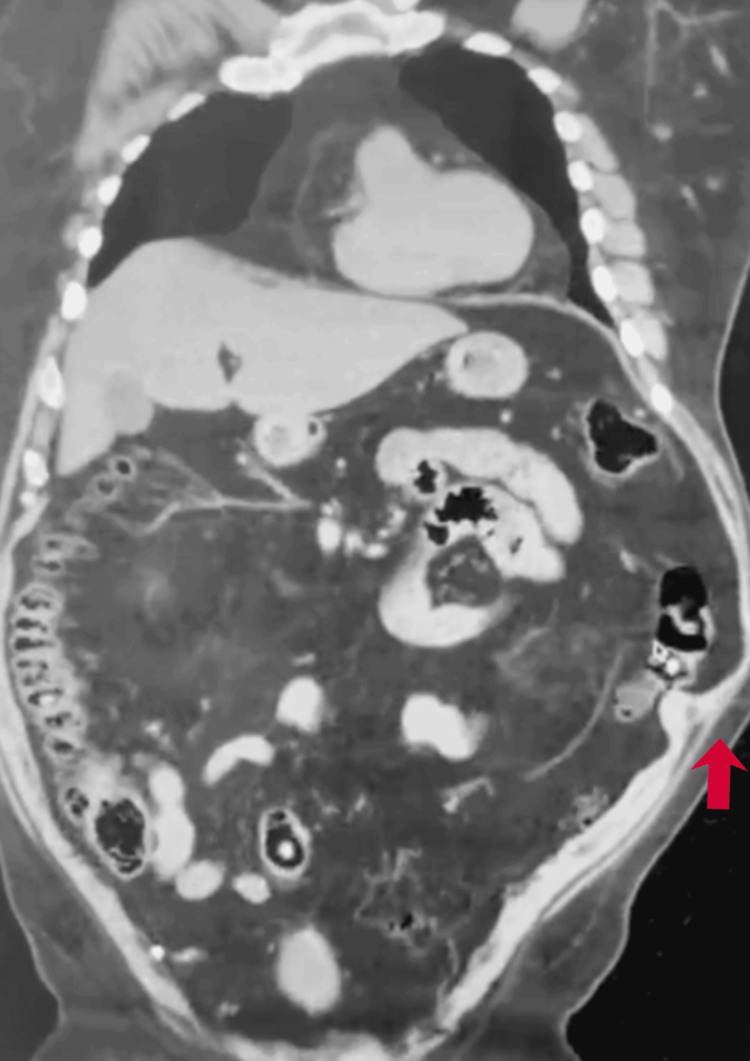
Coronal computed tomography image demonstrating the left lateral abdominal wall defect and protrusion of contents through the defect, with improved delineation during dynamic maneuvers

The absence of laboratory abnormalities (Table 1) and systemic signs, combined with a large but fully reducible defect without radiologic features of strangulation, supported elective repair in order to correct the structural defect of the lateral abdominal wall and prevent further progression or potential complications.

**Table 1:** Preoperative laboratory evaluation showed no significant hematologic, metabolic, or inflammatory abnormalities BUN: blood urea nitrogen

Parameter	Patient value	Reference range
Hemoglobin	13.4 g/dL	12.0-16.0 g/dL
Hematocrit	40.1%	36%-46%
Leukocyte count	7.8 × 10³/µL	4.0-10.0 × 10³/µL
Platelet count	248 × 10³/µL	150-400 × 10³/µL
Glucose	92 mg/dL	70-100 mg/dL
Lactate	0.7 mmol/L	0.5-2.0 mmol/L
Creatinine	0.82 mg/dL	0.6-1.2 mg/dL
BUN	14 mg/dL	7-20 mg/dL
Sodium	139 mmol/L	135-145 mmol/L
Potassium	4.1 mmol/L	3.5-5.0 mmol/L
Chloride	103 mmol/L	98-107 mmol/L
C-reactive protein	0.4 mg/dL	<0.5 mg/dL

Figure 3 presents the clinical examination image and the CT image.

**Figure 3:**
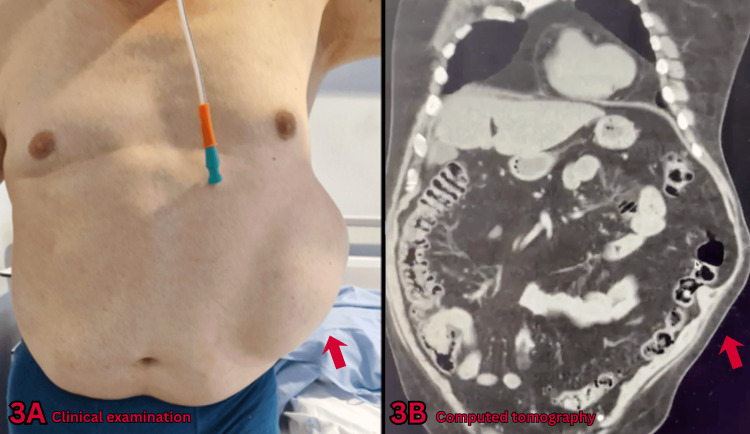
Clinicoradiological correlation showing clinical bulging of the left lateral abdominal wall alongside coronal computed tomography A: Clinical examination in the standing position during the Valsalva maneuver demonstrating a large abdominal wall hernia measuring approximately 25 cm in diameter. B: Computed tomography revealing a wide intercostal defect with herniation of intestinal loops, without radiologic evidence of incarceration or strangulation.

Therapeutic intervention

Following standard surgical preparation with appropriate aseptic and antiseptic technique, the procedure was performed under balanced general anesthesia with the patient in the supine position and slight left lateral tilt. After sterile draping, a supraumbilical vertical incision was made, and the subcutaneous tissue was dissected down to the aponeurosis.

A Veress needle was inserted, and pneumoperitoneum was established to 15 mmHg. A 10-mm trocar was placed in the epigastrium and an additional 10-mm trocar in the right flank. Laparoscopic exploration was performed, confirming the absence of iatrogenic injury at the access site. Adhesiolysis was subsequently carried out, and an intercostal hernia located between the eighth and ninth ribs was identified and reduced. The surrounding structures were inspected, confirming preservation of the abdominal wall without compromise of adjacent organs. Given these findings, conversion to the open portion of the procedure was undertaken.

An approximately 15-cm incision was then performed directly over the defect. Tissue planes were dissected until the hernia defect was fully exposed. Primary closure of the defect was subsequently achieved using a continuous Prolene 1-0 suture, reinforced with interrupted supporting stitches (Figure 4). Subsequently, laparoscopic intraperitoneal reinforcement was performed using V-Loc sutures (Figure 5). The integrity of the repair was verified, and skin closure was completed with staples.

**Figure 4:**
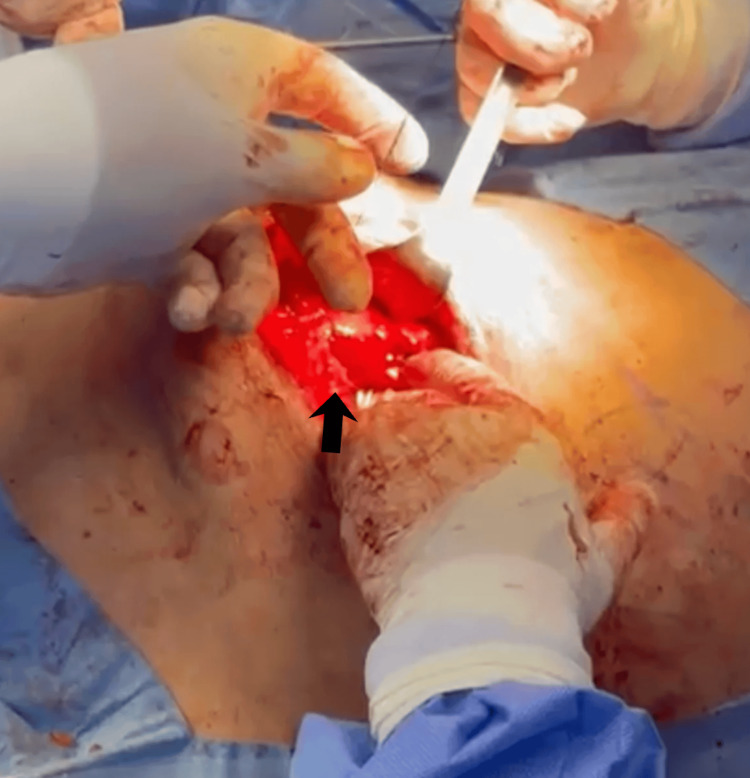
Intraoperative image demonstrating repair of the defect by primary closure

**Figure 5:**
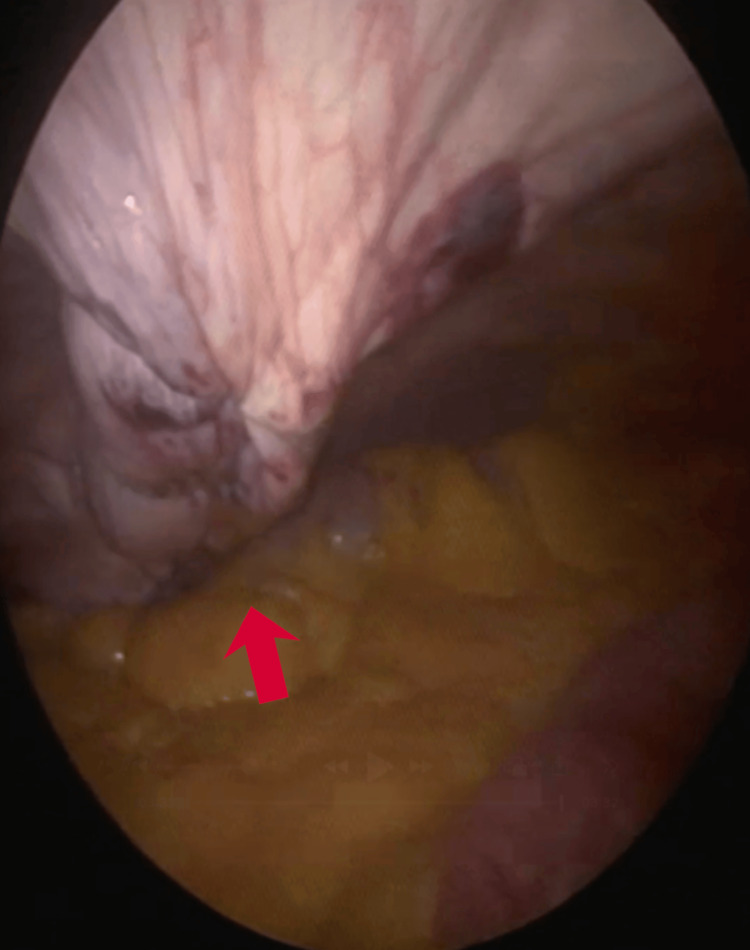
Intra-abdominal intraoperative image demonstrating laparoscopic reinforcement of the defect repair

The procedure concluded without complications, and the patient was transferred to the recovery unit in a stable condition.

The total operative time was 55 minutes with an uneventful postoperative course. The patient was subsequently discharged after 48 hours. At 60-day follow-up, the patient remained asymptomatic, without recurrence or chronic pain.

## Discussion

Intercostal hernias represent an uncommon clinical entity that has limited the development of standardized management guidelines and robust evidence-based recommendations. Most of the available literature consists of isolated case reports and small case series, making comparative analysis particularly important in understanding their pathogenesis and optimal treatment strategies.

Consistent with the systematic review published by Erdas et al., acquired intercostal hernias constitute the predominant subtype and are most frequently associated with previous surgery, trauma, or conditions causing chronic elevation of intra-abdominal pressure [1]. In contrast to traumatic presentations commonly described in the literature, the present case appears to be primarily related to prior surgical intervention, supporting the concept that disruption of the muscular and fascial integrity of the lateral abdominal wall may predispose patients to hernia formation. Furthermore, the involvement of the external oblique muscle observed intraoperatively in this patient correlates with previously described mechanisms of myofascial weakening and segmental denervation following abdominal wall procedures.

Moreno-Egea et al. highlighted that the lateral abdominal wall contains anatomically vulnerable regions extending between the costal margin and iliac crest, where the oblique and transversus muscle layers may become structurally compromised after surgical dissection or traumatic injury [2]. The anatomical findings in the present case closely resemble these observations, particularly regarding the progressive weakening of the lateral musculature and enlargement of the defect over time. Compared with lumbar hernias described in their series, however, the current defect demonstrated a more superior thoracoabdominal extension involving the intercostal space, emphasizing the spectrum and overlap between lateral abdominal wall and intercostal hernias.

Imaging findings in this case were also consistent with previous reports. Lee and Cohen emphasized the diagnostic value of computed tomography in accurately defining the size, location, and contents of abdominal wall hernias while excluding complications such as incarceration or strangulation [3]. In the present patient, computed tomography proved essential for preoperative planning by demonstrating intestinal herniation through the defect without signs of vascular compromise. Additionally, the use of dynamic clinical examination with the Valsalva maneuver allowed clearer characterization of the protrusion, findings that have been similarly described in prior imaging-based analyses.

Regarding treatment, there is general agreement that symptomatic intercostal hernias require surgical correction due to the risk of progressive enlargement, pain, incarceration, and impairment of abdominal wall function. Kurer and Bradford described successful laparoscopic repair in selected patients, highlighting the advantages of minimally invasive approaches, including reduced postoperative pain and shorter recovery time [4]. Nevertheless, large defects with extensive muscular disruption, such as in the present case, may require combined or open reconstructive techniques to achieve adequate reinforcement and restoration of abdominal wall integrity. Similarly, Gupta et al. reported that delayed management of defects secondary to trauma or surgery has a higher likelihood of progression when not treated promptly, especially in the presence of significant muscular weakness [5].

Overall, the present case reinforces the multifactorial pathophysiology of intercostal hernias and demonstrates the importance of correlating anatomical findings, imaging characteristics, and patient surgical history when planning treatment. In addition, it contributes to the limited existing literature describing postoperative lateral thoracoabdominal hernias and supports the need for individualized surgical strategies based on defect size, anatomical location, and degree of muscular involvement.

The limitation of our study is that follow-up is limited to 60 days and does not yet address longer-term recurrence risk.

## Conclusions

Intercostal hernias are rare entities in which computed tomography with the Valsalva maneuver plays a fundamental role in accurate diagnosis and appropriate surgical planning. In the present case, clinicoradiological correlation enabled precise identification of the defect and selection of an individualized combined laparoscopic and open approach, resulting in a favorable outcome without recurrence or chronic pain during initial follow-up. Due to the low incidence of this condition, there are currently no standardized guidelines or universally accepted gold standard techniques for repair; therefore, management should be individualized according to the anatomical and functional characteristics of each patient. Although much of the literature recommends the use of prosthetic mesh in large defects to reduce recurrence risk, this case suggests that reinforced primary closure may represent a safe and effective alternative in carefully selected scenarios, although longer follow-up and additional reports are needed to confirm durability.

In this patient, the absence of tension during closure, adequate tissue quality, lack of friability, and preservation of the costal wall without the need for rib approximation allowed successful reinforced primary repair and laparoscopic reinforcement with favorable clinical evolution. This report contributes to the limited available evidence regarding intercostal hernias and underscores the importance of tailoring the surgical strategy according to the specific characteristics of the defect and surrounding tissues.
